# Hyperfine Decoupling of ESR Spectra Using Wavelet Transform

**DOI:** 10.3390/magnetochemistry8030032

**Published:** 2022-03-08

**Authors:** Aritro Sinha Roy, Madhur Srivastava

**Affiliations:** 1 Department of Chemistry and Chemical Biology, Cornell University, Ithaca, NY 14853, USA;; 2 National Biomedical Center for Advanced ESR Technology, Cornell University, Ithaca, NY 14853, USA;

**Keywords:** ESR hyperfine decoupling, ESR resolution enhancement, wavelet transform, signal processing

## Abstract

The objective of spectral analysis is to resolve and extract relevant features from experimental data in an optimal fashion. In continuous-wave (cw) electron spin resonance (ESR) spectroscopy, both g values of a paramagnetic center and hyperfine splitting (A) caused by its interaction with neighboring magnetic nuclei in a molecule provide important structural and electronic information. However, in the presence of g - and/or A-anisotropy and/or large number of resonance lines, spectral analysis becomes highly challenging. Either high-resolution experimental techniques are employed to resolve the spectra in those cases or a range of suitable ESR frequencies are used in combination with simulations to identify the corresponding g and A values. In this work, we present a wavelet transform technique in resolving both simulated and experimental cW-ESR spectra by separating the hyperfine and super-hyperfine components. We exploit the multiresolution property of wavelet transforms that allow the separation of distinct features of a spectrum based on simultaneous analysis of spectrum and its varying frequency. We retain the wavelet components that stored the hyperfine and/or super-hyperfine features, while eliminating the wavelet components representing the remaining spectrum. We tested the method on simulated cases of metal-ligand adducts at L-, S-, and X-band frequencies, and showed that extracted g values, hyperfine and super-hyperfine coupling constants from simulated spectra, were in excellent agreement with the values of those parameters used in the simulations. For the experimental case of a copper(II) complex with distorted octahedral geometry, the method was able to extract g and hyperfine coupling constant values, and revealed features that were buried in the overlapped spectra.

## 1. Introduction

Continuous-wave electron spin resonance (cw-ESR) spectra of molecules with many interacting nuclei are often poorly resolved due to both line broadening and line splitting effects [1]. In many cases, determination of hyperfine coupling constants directly from cW-ESR spectra in an unambiguous manner are non-trivial and require sophisticated computational simulations [2,3]. In common practice, high-frequency and high field ESR spectroscopy is used for g-anisotropy resolution [4–7], while relatively low-frequency ESR is utilized in resolving hyperfine and/or super-hyperfine spectra [8,9] or a multi-frequency approach is utilized for analysis [10,11]. On the other hand, high resolution techniques, such as electron spin echo envelope modulation (ESEEM) and electron nuclear double resonance (ENDOR) spectroscopy, have been developed to resolve hyperfine interactions [1,12–17].

In this work, we present a wavelet transform technique [18] to decompose a cw-ESR spectrum into its hyperfine and super-hyperfine components or perform a *pseudo-decoupling* of the spectrum. We call it *pseudo-decoupling* because it does not decouple selected hyperfine interactions in a system in true sense, but it probes the spectra at various resolutions, enabling the separation of distinct features based on simultaneous analysis of magnetic field and its signal frequency at different resolutions. Wavelet transform calculates a pair of approximate and detailed spectra as a function of magnetic field and its signal frequency, effectively decoupling it into constituent components, including those that are overlapped. The Approximation and Detail wavelet components associated with hyperfine and/or super-hyperfine spectra can be singled out and then reconstructed in the spectral domain via inverse wavelet transform to obtain the desired feature. It is worth mentioning that wavelet transform has been previously used to extract ESR spectral parameters, albeit for a simpler case of X-ray irradiated guanidinium aluminium sulphate hexahydrate crystal [19]. The method used a first derivative of Lorentzian function as a wavelet basis to extract the spectral components, as ESR spectra is constituted from the Lorentzian function.

Using simulated and experimental data, we show that the *pseudo-decoupling* using wavelet transform can reliably resolve g- and A-anisotropy values of hypefine and superhyperfine lines. The resulting enhancement of spectral resolution reveals key features, improves accuracy of analysis and simplifies simulation efforts. More importantly, this simple add-on technique provides more control to ESR users by allowing them to focus on different aspects of the spin-system under study from the wavelet transform components of a spectrum. The presented signal processing tool can be used in conjunction with cw-ESR experiments and simulation for the extraction of an optimal amount of information from a given spectrum.

## 2. Materials

**Figure 1.**
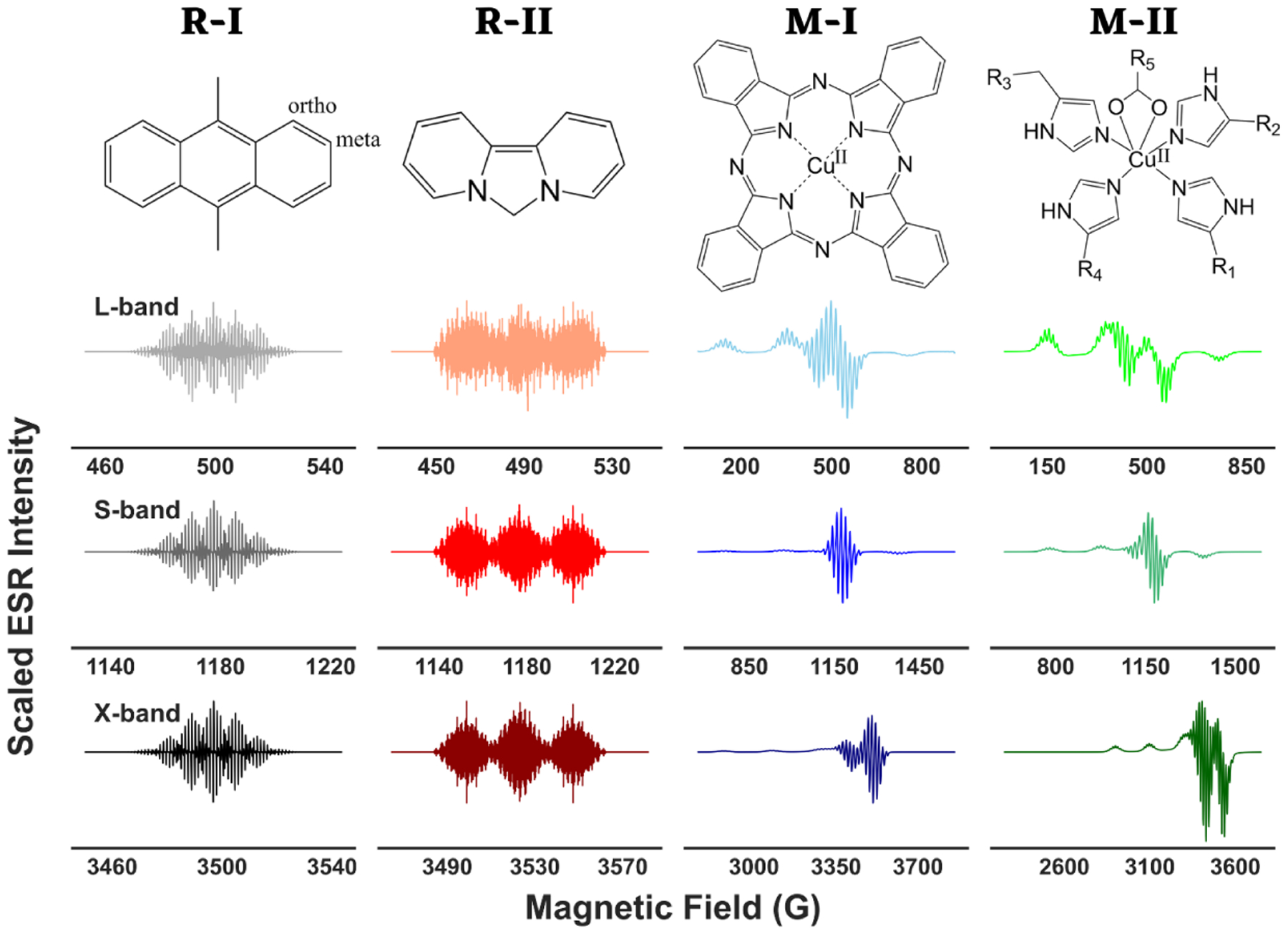
The organic radicals (R-I, R-II) and metal-ligand adducts (M-I, M-II) used in this work along with their simulated ESR spectra at L-, S-, and X-band frequencies.

We used simulated ESR spectra of two cation radicals, 9,10-dimethylanthracene (R-I) and 6-hydrodipyrido[1,2-c:2′, 1′-e]-imidazole (R-II), and two metal-ligand adducts, copper pthalycyanide or CuPc (M-I) and another copper-nitrogen adduct (M-II) [20] for illustration purposes, shown in Figure 1. An experimental ESR spectrum of a copper(II) complex with distorted octahedral geometry [21] was used.

### 2.1. Spin-Systems

We selected a pair of organic radicals and metal-ligand adducts in our analysis because they pose very different kinds of challenges. In case of organic radicals, the goal is to simplify the super-hyperfine spectra using wavelet transform in order to assign the hyperfine coupling constants accurately, while resolving the g- and A-anisotropy is often of major interest in the case of metal-ligand adducts [21,22].

R-I is a classic system in magnetic resonance studies [23], and its spectrum has 175 resonance lines. The unpaired spin in an R-I cation radical delocalizes onto the methyl groups [23,24] and hence the strongest hyperfine interactions caused by the six equivalent methyl protons split the central resonance line into seven lines. Four ortho-protons of the aromatic rings split each of those lines into five, followed by splitting of each lines into another five lines by the meta-protons. We simulated its isotropic spectra using the parameter values in Table 1 and a Gaussian lineshape of width 0.2 G at X-band. The linewidth at lower frequencies is expected to be smaller than that [25] and hence its value was set to 0.18 G and 0.17 G for the simulations at S- and L-band.

Next, we selected a bridged biaryl radical cation, R-II because of a large number of resonance lines (1215) in its ESR spectrum [26], which makes it an excellent case-study for our wavelet transform technique, and it falls into an important class of compounds for its relevance in the study of natural compounds. The lineshape was taken to be Lorentzian in these cases with peak-to-peak width of 0.06 G, 0.05 G, and 0.05 G at X-, S-, and L-band.

Copper plays an essential role in many catalytic processes, enzyme activities and electron-transfer processes [27–30]. The adducts, M-I and M-II, were chosen to be axially symmetric with a ${\;}^{63}\text{Cu}$ nucleus at the center, coordinated to four ${\;}^{14}\;\text{N}$ nuclei. In both the cases, the large $A_{∥}$ for copper, which has a nuclear spin of I=3/2, splits the resonance line corresponding to $g_{∥}$ into a quartet and the splitting of $g_{⊥}$ due to a relatively small $A_{⊥}$ of the copper nucleus is often not visible. Additionally, the super-hyperfine splitting of each line into nine lines by the ${\;}^{14}\;\text{N}$ nuclei makes the spectra in this region either poorly resolved due to overlapping lines or *noisy*. In both cases, determination of $g_{⊥}$ with high reliability or identification of minor differences in the value of $g_{⊥}$ or $A_{⊥}$ is a formidable task, especially at X-band or lower frequencies. In such cases, our objective is to decouple the hyperfine and super-hyperfine spectra to resolve the g- and A-anisotropy. The slow-motional spectra for M-I were calculated by treating ${\;}^{14}\;\text{N}$ nuclei perturbationally in the simulation software and the correlation time was set to $10^{-7.5}\;\text{s}$. In case of M-II, frozen cW-ESR spectra were simulated. Gaussian lineshape with varying widths of 3.0 G, 2.6 G, and 2.5 G was set at X-, S-, and L-band ESR simulations for M-I and for M-II, peak-to-peak width of Lorentzian lines was set to 6.5 G, 6.0 G, and 5.5 G, respectively.

### 2.2. Choice of Microwave Frequency

The simulations for all the systems were performed at L-, S-, and X-band. A virtue of the wavelet transform is that it produces a hyperfine component from a cW-ESR spectrum which resembles the corresponding high- to very high-field spectrum. Hence, it is only reasonable to use relatively low-to moderate-fields in our analysis. In addition to that, most ESR studies in applied fields are performed at X-band or lower fields because of higher accessibility and feasibility.

### 2.3. Simulation of ESR Spectra

Cw-ESR spectral simulations were carried out using EPRLL [31,32], EasySpin (version 5.2.33) [33], and Spinach [34] software, all of which are robust methods for generating such spectra and the open source code for each of them are readily available. For the cation radicals, isotropic cw-ESR spectra at L- (1.4 GHz), S- (3.3 GHz) and X-bands (9.8 GHz) were simulated, while slow-motional [31,32,34,35] and frozen spectra were calculated for M-I and M-II. The simulation parameters are tabulated in Table 1. All three software generated the same spectra. As the EasySpin is most widely used, we have provided the corresponding codes to generate the spectra in the Appendix A for the general audience.

## 3. Methods

### 3.1. Wavelet Transforms

Wavelet transform can decompose a cw-ESR spectra in the magnetic field-signal frequency domain, where different spectral features are stored in distinct wavelet components. This conveniently allows us to keep the wavelet components that contain hyperfine or super-hyperfine features, while eliminating the other wavelet components, or vice versa. Mathematically, a wavelet transform is defined as [36,37],

(1) $$F\left(\tau ,s\right)=\frac{1}{\sqrt{\left|s\right|}}\int_{-\infty }^{+\infty }f\left(B\right)\psi^{\text{*}}\left(\frac{B-\tau }{s}\right)dt$$

where s is the inverse frequency (or frequency range) parameter, τ is the signal localization parameter, $B_{0}$ represents the magnetic field location, f(B) is the spectrum, F(τ,s) is the wavelet-transformed signal at a given signal localization and frequency, and $\psi^{*}\left(\frac{B-\tau }{s}\right)$ is the signal probing function called “wavelet”. Different wavelets are used to vary selectivity or sensitivity of adjacent frequencies with respect to signal localization. They are not dependent on a priori information of the signal or its characteristics. Depending on the application, different wavelets can be selected.

In discrete form, wavelet transform is expressed by two sets of wavelet components (Detail and Approximation) in the following way [36]:

(2) $$D_{j}\left[n\right]=\overset{p-1}{\underset{m=0}{\sum }}f\left[B_{m}\right]2^{\frac{j}{2}}\psi \left[2^{j}B_{m}-n\right]$$

and

(3) $$A_{j}\left[n\right]=\overset{p-1}{\underset{m=0}{\sum }}f\left[B_{m}\right]2^{\frac{j}{2}}\phi \left[2^{j}B_{m}-n\right]$$

where $f\left[B_{m}\right]$ is the discrete input signal, p is the length of input signal $f\left[B_{m}\right],D_{j}[n]$, and $A_{j}[n]$ are the Detail and Approximation components, respectively, at the jth decomposition level, and $\psi \left[2^{j}B_{m}-n\right]$ and $\phi \left[2^{j}B_{m}-n\right]$ are wavelet and scaling functions, respectively. The maximum number of decomposition levels that can be obtained is N, where $N={\text{log}}_{2}\;p$, and 1≤j≤N. The scaling and wavelet functions, at a decomposition level, are orthogonal to each other, as they represent non-overlapping frequency information. Similarly, wavelet functions at different decomposition levels are orthogonal to each other.

The Detail component $D_{j}[n]$ is the discrete form of Equation (1), where j and n are associated with s and τ, respectively. The Approximation component $A_{j}[n]$ represents the remaining frequency bands not covered by the Detail components till the jth level. The signal $f\left[B_{m}\right]$ can be reconstructed using the inverse discrete wavelet transform as follows:

(4) $$f\left[B_{m}\right]=\overset{p-1}{\underset{k=0}{\sum }}A_{j_{0}}\left[k\right]\phi_{j_{0},k}\left[B_{m}\right]+\overset{j_{0}}{\underset{j=1}{\sum }}\overset{p-1}{\underset{k=0}{\sum }}D_{j}\left[k\right]\psi_{j,k}\left[B_{m}\right]$$

where $j_{0}$ is the maximum decomposition level from which an input signal needs to be reconstructed.

### 3.2. NERD Method

We use Noise Elimination and Reduction via the Denoising (NERD) method [38], which is based on wavelet transforms, to identify and to separate the hyperfine and super-hyperfine lines. The NERD method is primarily used for denoising 1D signals, including cW-ESR spectra, but we adapt it for the purpose of hyperfine feature extraction. We then have the following steps:

#### (a) Wavelet Selection:

There are many standard wavelet families that can be used for the UDWT. We use the Daubechies family wavelet “db6”. The Daubechies family provides the best sensitivity and selectivity for adjacent frequency values, as it maximizes the vanishing moments. Better frequency resolution is essential for distinguishing and separating features from the overlapped spectra. db6 wavelet is selected for its appropriate length. A smaller length may not capture all the necessary information, whereas a larger length would yield redundant information.

#### (b) Undecimated Discrete Wavelet Transform:

In NERD, we use the undecimated discrete wavelet transform [39] to achieve the maximum signal resolution in the Detail and Approximation components. This means that each Detail and Approximation component has the same length as that of the input signal. For instance, a signal with data length of 1024 will have 10 Detail and Approximation components (from 10 decomposition levels), each having a length of 1024 data points. The maximum number of decomposition levels, N, is defined as $N={\text{log}}_{2}\;p\;(\;p$ being the input signal length) [40]. The UDWT improves the resolution in the wavelet domain by preserving the input data length at all decomposition levels. In the decimated version, the Detail and Approximation components are downsampled by 2 at each level, and hence reduces the resolution essential for extracting the hyperfine lines.

#### (c) Detail Component Selection:

This step is adapted in the NERD method for the feature extraction purpose. For denoising, this step is associated with identifying noisy Detail components and applying noise thresholds to remove it [18,38,41,42]. For feature extraction, we retain the Detail components that contain hyperfine and/or super-hyperfine spectra, while removing all other Detail components, including the Approximation component. This step is repeated for each feature.

The NERD software can be accessed through denoising.cornell.edu (accessed on 1 January 2022), which is available since 20 February 2019. The algorithm (cf. Algorithm 1) for the method is given below:

**Algorithm 1:** Adapted NERD Algorithm for pseudo-decoupling

1:	Select db6 wavelet.
2:	Apply undecimated discrete wavelet transform.
3:	Select *N* decomposition levels to obtain all the Detail and Approximation components, where *N* = ⌊log_2_(*Signal*_*Length*_)⌋.
4:	Retain the Detail components associated with the desired feature and remove all the values of the remaining Detail components.
5:	Remove all the values of the *N*th Approximation components.
6:	Take the inverse undecimated discrete wavelet transform of the resultant *N* Detail components and the *N*th Approximation component.

## 4. Results and Discussion

### 4.1. Analysis of Isotropic ESR Spectra

**Figure 2.**
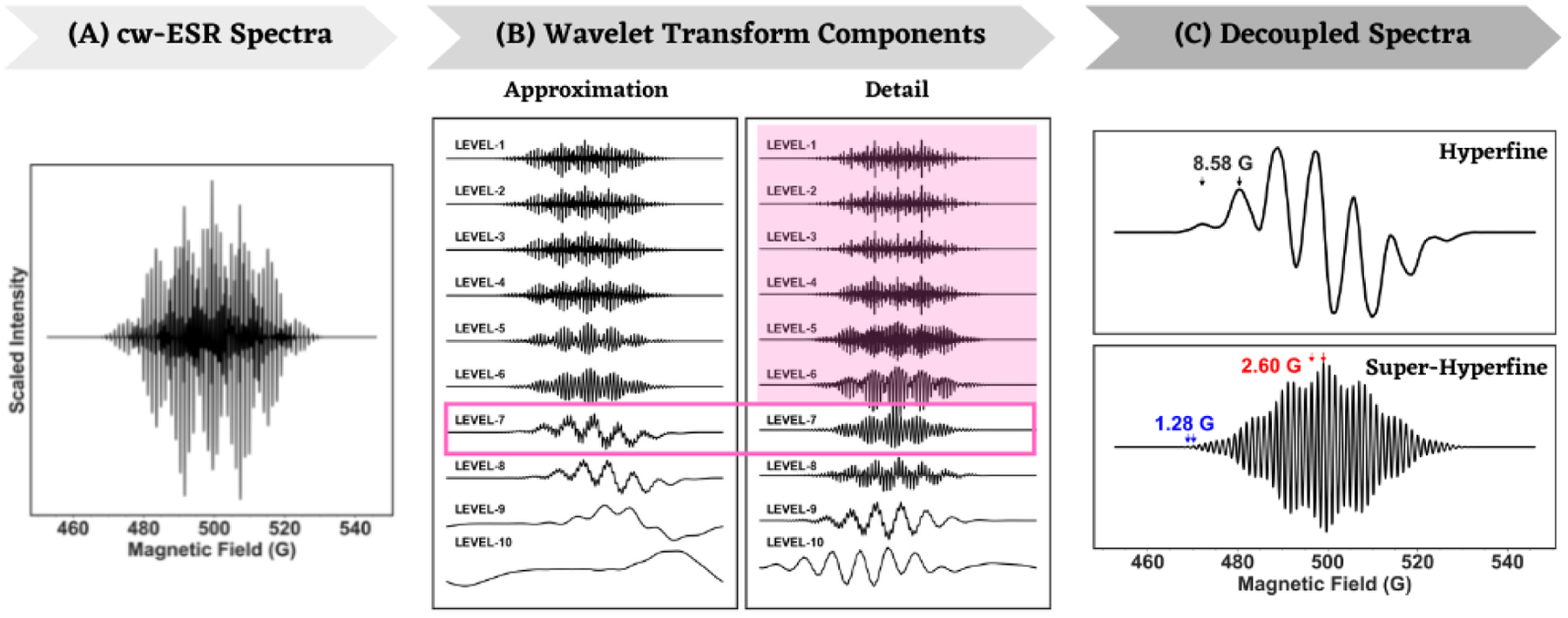
Simulated L-band cW-ESR spectra of R-I (**A**), wavelet transform Approximation and Detail components at different levels (**B**) and separation of the hyperfine and super-hyperfine components (**C**). The detailed component was removed from the spectra at each level of the analysis till level-6 (magenta background) and complete separation of hyperfine and super-hyperfine components occurred at level-7 in (**B**) (magenta box). Colored pair of arrows and text in (**C**) indicate resolved hyperfine and/or super-hyperfine splittings.

A cw-ESR spectrum of R-I has 175 resonance lines. Many of those lines overlap and hence the resulting modified intensities of the lines make the assignment of hyperfine splitting even harder. A simulated isotropic L-band ESR spectrum of R-I is shown in Figure 2A and the corresponding hyperfine and super-hyperfine components post wavelet transform are shown in Figure 2C. The approximate and detailed components at different levels of wavelet transform are shown in Figure 2B, and it is evident from the plots that a complete separation of the hyperfine and super-hyperfine components occurs at level-7. Beyond level-8, the components did not provide any useful information and, by level-10, entire spectral information was lost [42]. Hence, the detailed components from level-1 through level-6 were removed from the spectra sequentially and the decomposition at level-7 was used for the spectral analysis. The resulting hyperfine spectrum in Figure 2C was obtained by subtracting the detailed spectra at level-7, while subtraction of the approximate spectra yielded the super-hyperfine spectrum. The hyperfine spectral component shows splitting of the primary ESR line by six equivalent methyl-group protons into seven resonance lines. The relative intensities of super-hyperfine splittings by the ortho- and meta-protons are readily resolved in the super-hyperfine component in Figure 2C. In general, all the peak-to-peak splittings in the super-hyperfine component of a spectrum are calculated and the peaks with the same or very similar splittings are placed together in a bin, followed by mapping of each of the bins to super-hyperfine splittings by the various nuclei. Only a pair of peaks from each such bins are shown in any of the figures for convenience. The values obtained for R-I, $A_{\text{methyl }}=8.58\;\text{G},A_{\text{ortho-H }}=2.60\text{G},A_{\text{meta-H }}=1.28\text{G}$, are in good agreement with the values used in the simulation of the spectrum (7.91G,-2.49G,-1.20G). Analyses of S- and X-band spectra of R-I predicted similar values for the hyperfine coupling constants. While the analysis presented here used spectral decomposition at level-7, the decomposition at level-8 might appear to be optimal by visual inspection of the Approximation and Detail components. However, the optimal level of decomposition was chosen based on the resultant spectrum, obtained by iterative subtraction of Detail components from the original spectrum. Additionally, the optimal level of decomposition remained the same for a particular compound across all the ESR frequencies in all the analysis presented in this work.

**Figure 3.**
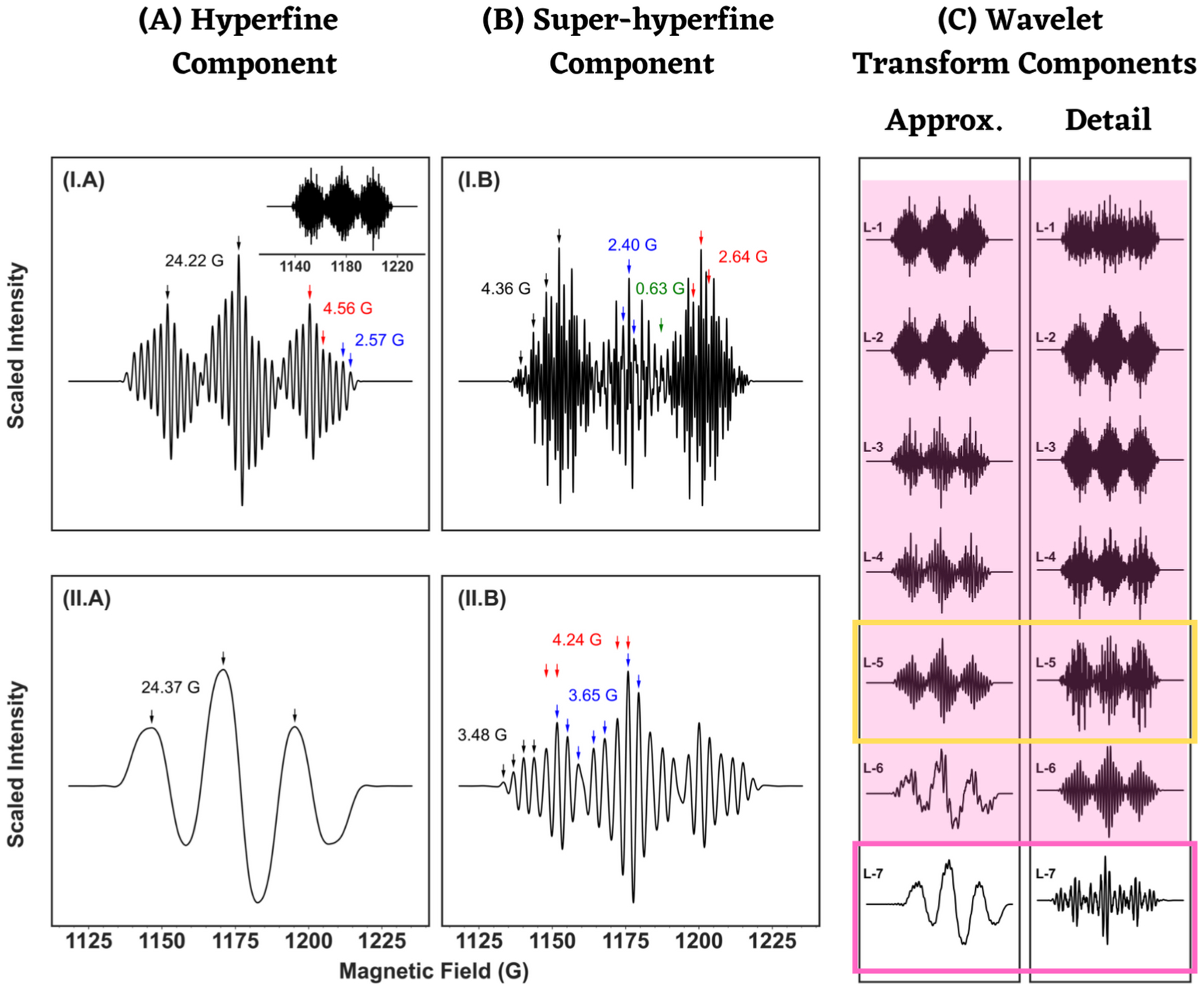
Separation of hyperfine (**A**) and super-hyperfine (**B**) components of an ESR spectrum of R-II at S-band. Figure (**I.A**) inset shows the simulated ESR spectra. Wavelet transform analysis from level-1 to level-7 is shown (**C**). Complete separation of hyperfine and super-hyperfine components occurred at level-7 (magenta box) after removing the detail component in each level of the wavelet transform application (magenta background). Much of the super-hyperfine coupling information was lost by level-7, while partial separation at level-5 (yellow box) was appropriate in resolving the super-hyperfine components. Resultant spectral components at both level-5 (I) and level-7 (II) are shown. The colored pair of arrows and text in (**A,B**) indicate resolved hyperfine and/or super-hyperfine splittings.

R-II has a much more complicated ESR spectra compared to R-I and its S-band spectrum is shown in the inset of Figure 3(I.A). Only the large hyperfine splitting by the ${\text{CH}}_{2}$-hydrogens could be determined directly from the original spectrum [26]. The wavelet transform hyperfine spectrum, Figure 3(I.A), retains the primary spitting by the ${\text{CH}}_{2-}$hydrogens as well as interaction with nitrogen $\left({\;}^{14}\;\text{N}\right)$ and an average interaction with the $\left(3,3^{\prime }\right)$ and $\left(5,5^{\prime }\right)$ ring-protons. The latter splittings were better resolved in Figure 3(I.B) and the small coupling of 0.65 G to the $\left(4,4^{\prime }\right)$ protons were resolved as well. The Approximation and Detail wavelet components at different levels are shown in Figure 3C. The analysis presented in (I.A) and (I.B) of the figure was performed at level-5, though complete separation of hyperfine and super-hyperfine components occurred at level-7. At level-7, most of the small super-hyperfine splitting information was removed by the analysis (II.A, II.B). The lower-level analysis was suited for the resolution of super-hyperfine spectral components. This is a key achievement of the analysis, which provides a user with access to different spectral features at different levels of the wavelet transforms. At present, the decomposition levels are visually selected. For the purpose of convenience and automation, finding the optimal level of decomposition via a set of objective criteria can be developed, but it is a part of another work. The values obtained for the various spectral parameters at L-, S-, and X-band frequencies by the wavelet transform method were comparable, demonstrating the generality of the method.

### 4.2. Analysis of Anisotropic ESR Spectra

**Figure 4.**
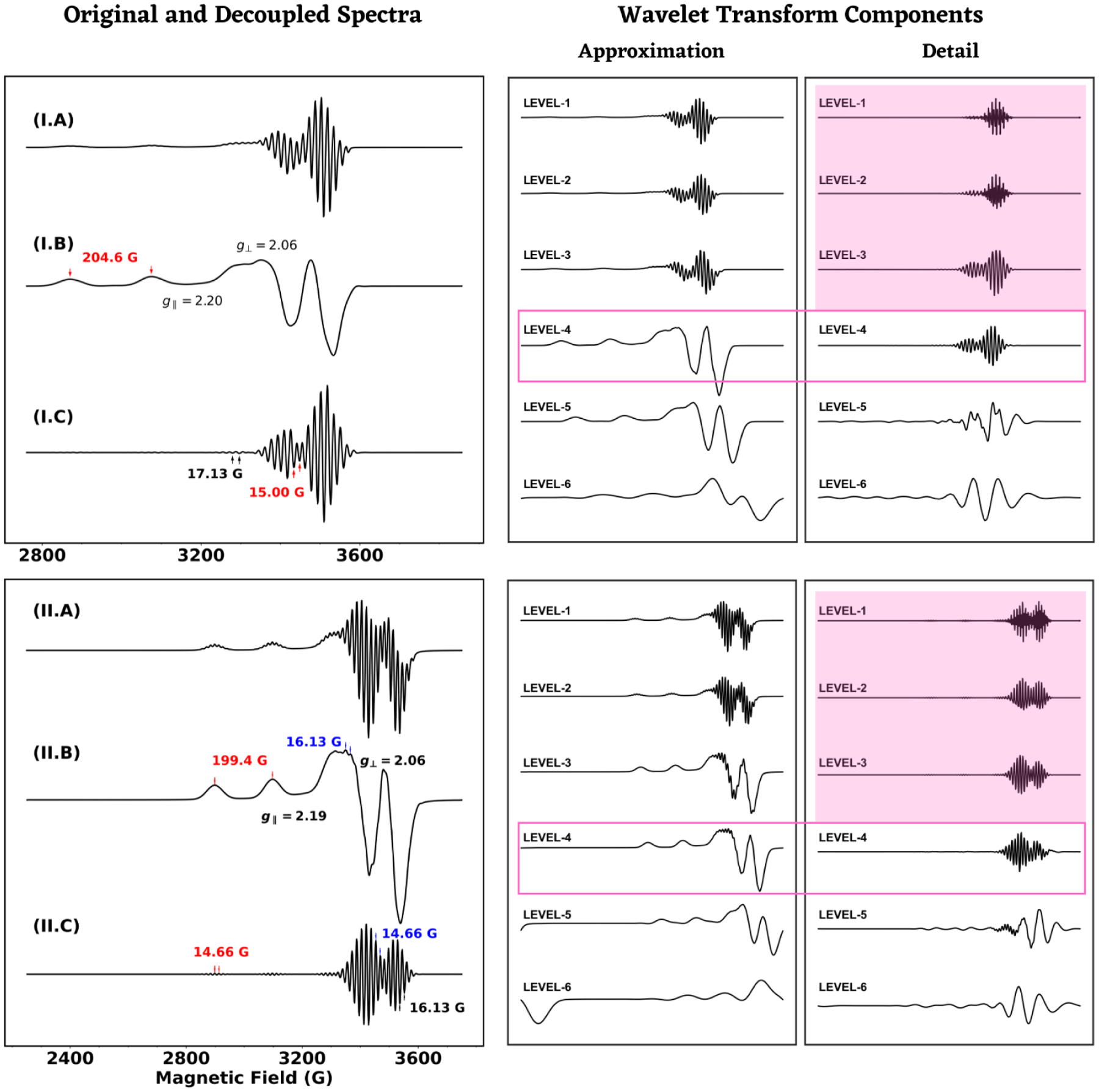
Original X-band cw-ESR spectra (left panel) and the corresponding decoupled components (right panel) for M-I (I) and M-II (II) are shown in the left panel, while the right panel shows wavelet transform components at different levels. The Detail components between level-1 and -3 were removed from the spectra sequentially (magenta background) and the resulting components at level-4 (magenta box) were used for both of the cases to perform the pseudo-decoupling. The values of $g_{∥}$ and $g_{⊥}$ were resolved along with the ${\;}^{14}\;\text{N}$ super-hyperfine coupling. Pairs of colored arrows are used to indicate hyperfine and super-hyperfine splittings.

Both of the metal-complexes, M-I and M-II, have four ${\;}^{14}\;\text{N}$-ligands attached to a copper ion, Cu(2+), in the center, and both adducts have an axial g-tensor with principal values of $g_{⊥}=2.05$ and $g_{∥}=2.19$ and 2.20, respectively. Spectral resolution beyond $g_{∥}$ and the very large parallel component of $A_{\text{Cu}}(\sim 200\text{G})$ was not possible from overall cW-ESR spectra of M-I and M-II, making determination of $g_{⊥}$ and $A_{\text{N}}$ with considerable accuracy a tricky task. Pseudo-decoupling of the X-band spectra of M-I and M-II is shown in Figure 4. At X-band and in the slow-motional regime, the spectral decomposition for M-I resolved both $g_{∥}$ and $g_{⊥}$, and the perpendicular component of $A_{\text{Cu}}$ and $A_{\text{N}}$ (error of 2% to 4%). The corresponding wavelet transform analysis of the ESR spectrum of M-II identified an average $A_{\text{N}}$ of 14.66 G in the low-field super-hyperfine spectra in Figure 4C and the splitting at high-field was determined to be 16.13 G. Compared to the simulation parameter values, the latter estimation differed only insignificantly. It should be noted that the predicted values could be optimized by fitting the original spectra using the method of least-squares or other optimization algorithms. We note that the resolution of the L-band spectra of M-I and M-II was much lower compared to that of S- and X-band frequencies, but the summary in Table 2 demonstrates consistency of the wavelet transform method in analyzing cW-ESR spectra across varying frequencies and resolutions.

### 4.3. Spectral Analysis of Experimental Data

**Figure 5.**
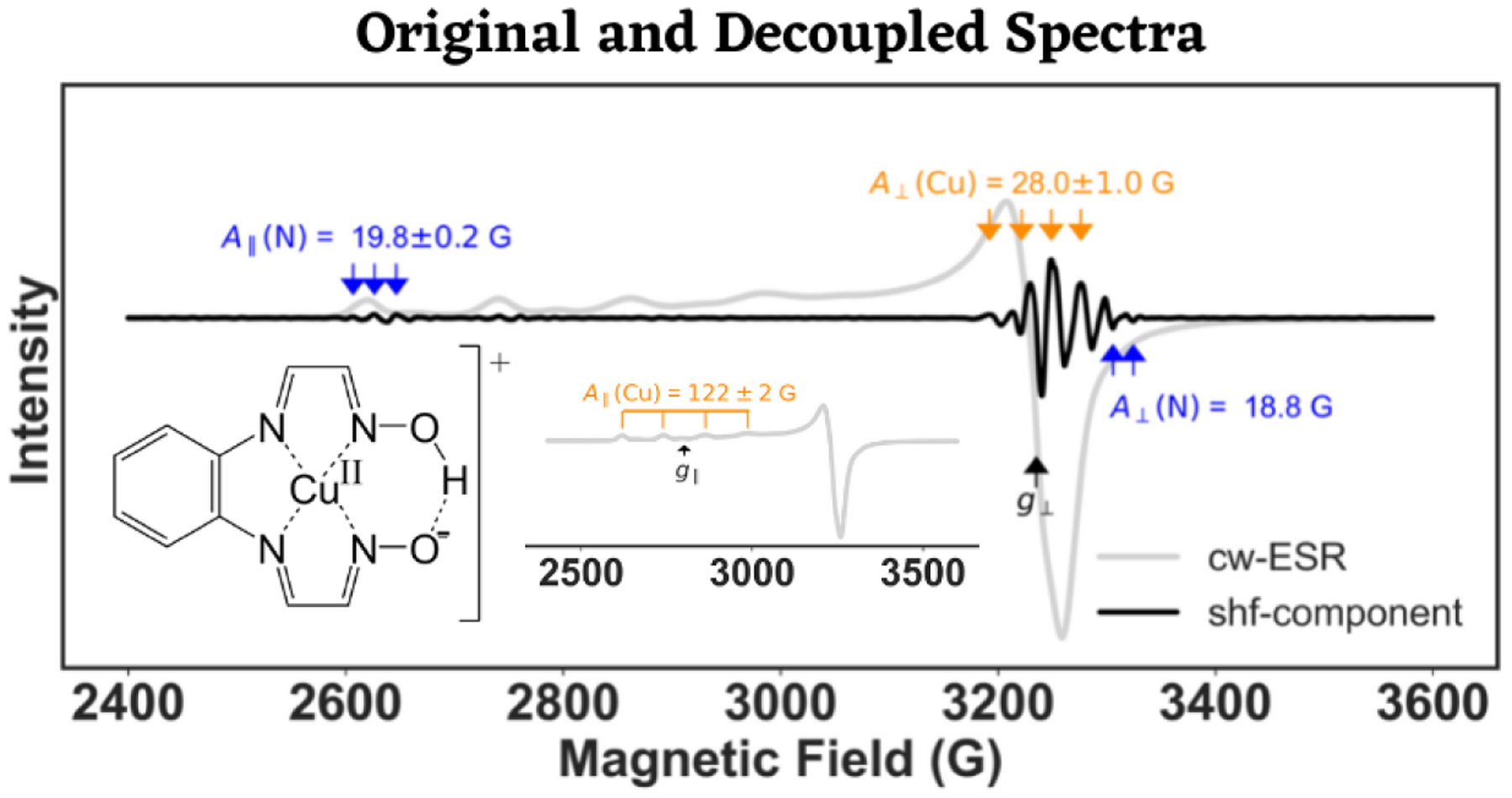

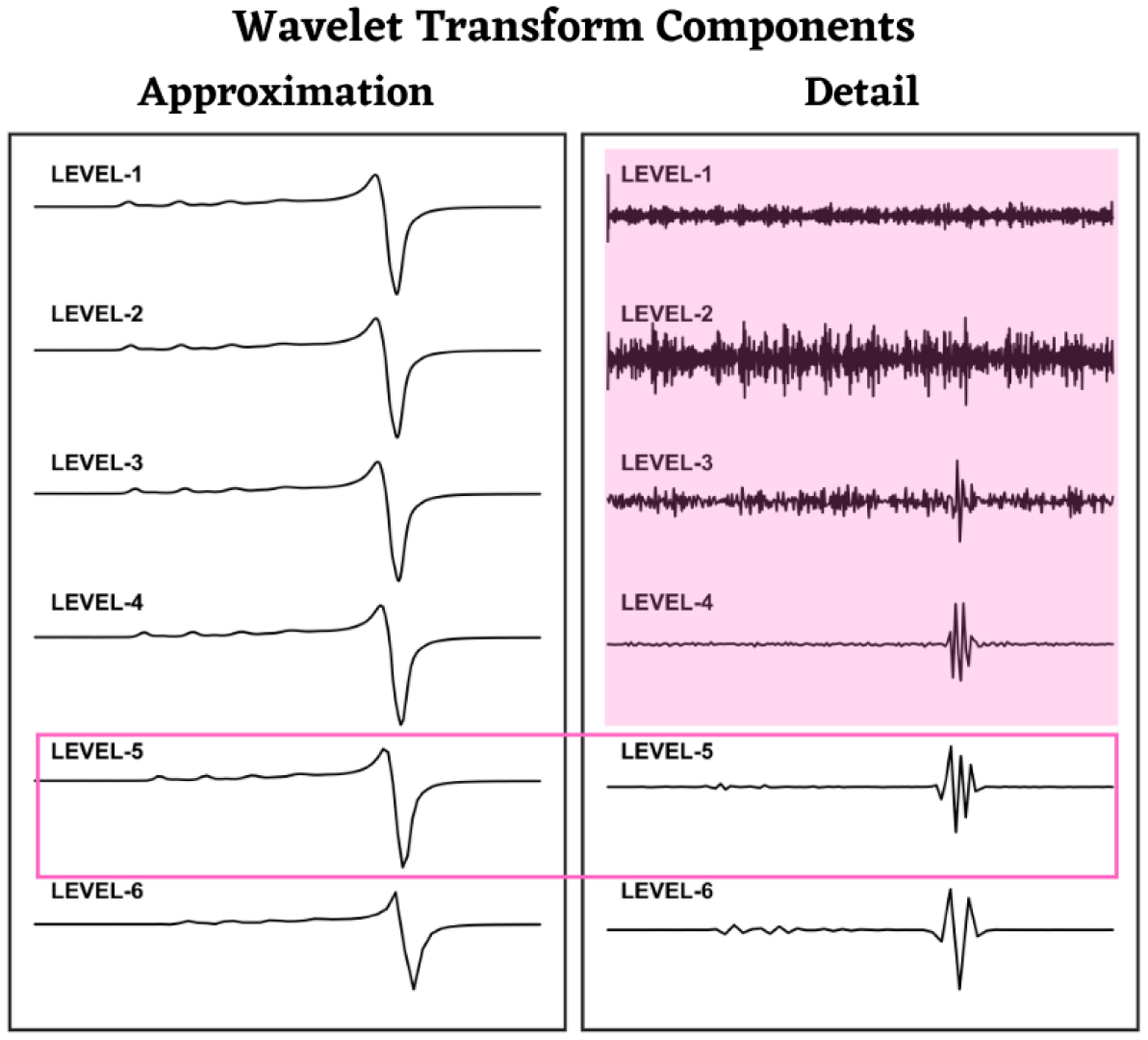
CW-ESR spectrum (light grey) of a $\text{Cu}(\text{II})-{\text{N}}_{4}$ complex (inset) and its wavelet transform super-hyperfine (shf) component (black) are shown in the top panel. The bottom panel shows the Approximation and Detail components of the analysis for level-1 to level-6, the optimal level of decomposition being level-5 (magenta box). From level-1 to −4, the Detail component was removed from the signal at each level of analysis. The values of $g_{∥}$ and $g_{⊥}$ were calculated to be 2.42 and 2.10, respectively, while $A_{∥}(\text{Cu})$ and $A_{⊥}(\text{Cu})$ were found to be 122.0±2G and 28.0±1.0G. The nitrogen super-hyperfine splitting constants, $A_{∥}\left(\text{N}\right)=19.8\pm 0.2\;\text{G}$ and $A_{⊥}\left(\text{N}\right)=18.8\text{G}$, were also resolved.

Experimental ESR spectra are often poorly resolved compared to the simulations illustrated so far. The experimental X-band cw-ESR spectrum of a $\text{Cu}(\text{II})-{\text{N}}_{4}$ complex is shown in Figure 5, and the structure of the complex is given in the inset of the top panel of the figure. From the original spectrum, the values of only $g_{∥}$ and $A_{∥}(\text{Cu})$ could be obtained, shown in Figure 5-inset. The authors of [21] determined the value of $g_{⊥}$ to be approximately 2.08, while $g_{⊥}$ was calculated to be 2.10 in a definitive manner by our analysis. The perpendicular hyperfine coupling constant of Cu(II) was found to be 28±1G and both of the nitrogen super-hyperfine interactions were resolved with reasonable certainty as well from the wavelet transform Detail component of the spectrum. It should be noted that the experimental spectra had a finite amount of noise, which can be seen in the detail component of the wavelet transform spectra in the bottom panel of Figure 5. The super-hyperfine splitting was invisible at level-1 and -2, owing to the presence of the noise. However, elimination of the Detail components from the spectrum up to level-3 and −4 revealed the splitting constants in the super-hyperfine spectral component. The subtraction of Detail components was continued to the point where the noise was completely removed from it, which was level-5 in this case.

## 5. Conclusions and Future Work

We presented a wavelet transform technique for hyperfine pseudo-decoupling of cw-ESR spectra at X-band and lower frequencies. The separation of a cw-ESR spectrum into its hyperfine and super-hyperfine components, which enhances spectral resolution significantly, is the core working principle of the technique. Analysis of simulated spectra to recover g and/or A values from both organic radicals and metal-ligand complexes is presented as the proof of concept. In all the cases, prediction of spectral parameters by our analysis matched reasonably well with the values used in the simulations across different operating ESR frequencies between 1.4 and ~ 9.9 GHz with varying widths for resonance lines. While the technique helps assignment of hyperfine splitting in the ESR spectrum of both organic radical and metal-ligand complexes, we believe that determination of g- and A-anisotropy of the latter could be the high-impact application of the technique in the near future. The hyperfine components produced by the wavelet transform of S- or X-band ESR spectra resemble the corresponding spectra at high to very high fields, which is less accessible to the primary users of ESR spectroscopy, and our method could bridge that gap in certain cases. A major difference between simulated and real spectra is that the super-hyperfine lines are often poorly resolved in the latter. We applied our method on an experimental X-band spectrum of a complex with $\text{Cu}-{\text{N}}_{4}$ core and successfully resolved the copper and nitrogen super-hyperfine splittings, while locating the position of $g_{⊥}$ without any ambiguity. Based on these results, we conclude that the wavelet transform technique has the potential to significantly reduce uncertainty in structural analysis of metal-ligand complexes by cw-ESR.

We plan to build a library of ESR experimental spectra and the corresponding wavelet transform analysis, with an emphasis on optimizing extraction of unresolved g- and A-anisotropy and/or unresolved super-hyperfine coupling constants. In addition to that, the library would significantly contribute to structural analysis of various molecules, especially metal-ligand adducts and metal-proteins by cw-ESR spectroscopy.

## Figures and Tables

**Table 1.** Simulation parameters for M-I, M-II, R-I, and R-II.

Molecule	Simulation Type	g Value	A (Gauss)
R-I	Isotropic	2.0316	$A_{\text{methyl }}=7.91$ $A_{\text{ortho }-\text{H}}=-2.49,A_{\text{meta }}-\text{H}=-1.20$
R-II	Isotropic	2.0316	$A_{\text{H}4}=-2.39,A_{\text{H}5}=-0.65,A_{\text{H}6}=-0.23$ $A_{\text{H}4}=-2.39,A_{\text{H}5}=-0.65,A_{\text{H}6}=-0.23$
M-I	Slow-motional	(2.05, 2.20)	$A_{\text{Cu}}=(-18.82,-18.82,-197.46)$ $A_{\text{N}}=(14.64,14.64,16.89)$
M-II	Frozen	(2.05, 2.19)	$A_{\text{Cu}}=(18.60,18.60,198.68)$ $A_{\text{N}}=(14.99,14.99,17.78)$

**Table 2.** Summary of spectral analysis by wavelet transform.

Molecule	L-Band	ESR Frequency S-Band	X-Band
R-I	$A_{\text{methyl }}=8.58$	8.52	8.20
	$A_{\text{O}-\text{H}}=2.60$	2.59	2.59
	$A_{\text{m}-\text{H}}=1.28$	1.28	1.28
R-II	$A_{\text{CH}2}=24.26$	24.22	24.22
	$A_{\text{N}}=4.34$	4.36	4.37
	$A_{\text{H}3,4,5}=2.63,2.46,0.83$	2.64, 2.40, 0.63	2.66, 2.51, 0.66
M-I	$g_{\|}=2.20,g_{⊥}=2.10\pm 0.02$	2.20, 2.02	2.20, 2.06
	$A_{\text{Cu}}=207.3$	207.4	204.6
	$A_{\text{N}}=14.98,16.65$	15.07, 15.96	15.00, 17.13
M-II	$g_{\|}=2.14,g_{⊥}=2.09\pm 0.02$	2.19, 2.01	2.19, 2.06
	$A_{\text{Cu}}=212.0$	200.4	199.4
	$A_{\text{N}}=14.96,15.84$	14.66, 16.62	14.66, 16.13

## Data Availability

The simulated data used in this paper can be accessed via https://github.com/Signal-Science-Lab and https://signalsciencelab.com/resources/, which is available since 26 December 2021. The NERD software is available via denoising.cornell.edu and https://signalsciencelab.com/resources/, which is available since 20 February 2019.

## Appendix A. EasySpin Codes

### Appendix A.1. Simulation Code for R-I ESR Spectra

A = mt2mhz([0.791, −0.249, −0.120], gfree); % conversion to MHz
Sys = struct(‘g’, gfree, ‘Nucs’, ‘1H,1H,1H’, ‘n’, [6, 4, 4],
‘A’, A, ‘lw’, 0.02);
Params = struct(‘mwFreq’, 9.8, ‘nPoints’, 8192);
% Simulation of isotropic spectra %
[B,spec] = garlic(Sys, Params);

### Appendix A.2. Simulation Code for R-II ESR Spectra

A_Gauss = [4.34, −2.39, −0.65, −2.81, −0.23, 24.24]; % in Gauss
A_MHz = mt2mhz(A_Gauss/10, gfree); % Gauss to MHz
Sys = struct(‘g’, 2.00316, ‘Nucs’, ‘14N,1H,1H,1H,1H,1H’,
‘n’, [2, 2, 2, 2, 2], ‘A’, A_MHz, ‘lwpp’, [0 0.006]);
Params = struct(‘mwFreq’, 9.878, ‘CenterSweep’, [352 24],
‘nPoints’, 4096);
% Simulation of isotropic spectra %
[B, spec] = garlic(Sys, Params);

### Appendix A.3. Simulation Code for M-I ESR Spectra

A_MHz = [−54 −54 −608; 52 42 42]; % in MHz
Sys = struct(‘g’, [2.0525 2.1994], ‘Nucs’, ‘63Cu,14N’,
‘n’, [1, 4], ‘A’, A_MHz, ‘tcorr’, 10^−7.5, ‘lw’, 0.3);
Params = struct(‘mwFreq’, 9.8);
% GHz
% Following setting is for treating the 14N nuclei
% perturbationally using post-convolution.
Opt.PostConvNucs = 2;
Opt.LLKM = [16 0 0 4]; % axial symmetry of 63Cu+e system
% Simulation of slow-motional spectra %
[x,y] = chili(Sys, Params, Opt);

### Appendix A.4. Simulation Code for M-II ESR Spectra

A_Cu = [0.0019 0.0203]; % in cm^−1
A_N = [0.00145 0.0017]; % in cm^−1
A_Frame = [0 0 0; 0 −1 0; −1 −1 0; −2 −1 0; −3 −1 0]*pi/2;
A_MHz = [A_Cu;A_N;A_N;A_N;A_N]*30e3; cm^−1 to MHz
Sys = struct(‘g’, [2.05 2.19], ‘Nucs’, ‘63Cu,14N,14N,14N,14N’,
‘A’, A_MHz, ‘AFrame’, A_Frame, ‘lwpp’, [0 0.65]);
Params = struct(‘mwFreq’, 9.8, ‘Range’, [225 375]);
Opt.Method = ‘perturb’;
Opt.nKnots = [181 0];
% Simulation of frozen spectra %
[x,y] = pepper(Sys, Params, Opt);

## Abbreviations

ESR: Electron spin resonance
PDS: Pulsed dipolar spectroscopy
NERD: Noise Elimination and Reduction via Denoising
